# Heart regeneration for clinical application update 2016: from induced pluripotent stem cells to direct cardiac reprogramming

**DOI:** 10.1186/s41232-016-0028-z

**Published:** 2016-10-24

**Authors:** Hiroyuki Yamakawa

**Affiliations:** 1 Department of Clinical and Molecular Cardiovascular Research, Keio University School of Medicine, Shinjuku-ku, Tokyo, Japan; 2 Department of Cardiology, Keio University School of Medicine, 35 Shinanomachi, Shinjuku-ku, Tokyo, 160-8582 Japan

**Keywords:** Cardiomyocytes, Cardiac fibroblasts, Myocardial infarction, Transcription factors, microRNAs, Cardiac regeneration, Induced cardiomyocytes, Direct reprogramming, iPS cells

## Abstract

Cardiovascular disease remains a major cause of death for which current therapeutic regimens are limited. Following myocardial injury, endogenous cardiac fibroblasts, which account for more than half of the cells in the heart, proliferate and synthesize extracellular matrix, leading to fibrosis and heart failure. As terminally differentiated cardiomyocytes have little regenerative capacity following injury, the development of cardiac regenerative therapy is highly desired. Embryonic stem and induced pluripotent stem (iPS) cells are promising tools for regenerative medicine. However, these stem cells demonstrate variable cardiac differentiation efficiency and tumorigenicity, which must be resolved prior to clinical regenerative applications. Until the last decade, an established theory was that cardiomyocytes could only be produced from fibroblasts through iPS cell generation. In 2010, we first reported cardiac differentiation from fibroblasts by direct reprogramming, and we demonstrated that various cardiac reprogramming pathways exist.

This review summarizes the latest trends in stem cell and regenerative research regarding iPS cells, a partial reprogramming strategy, and direct cardiac reprogramming. We also examine the many recent advances in direct cardiac reprogramming and explore the suitable utilization of these methods for regenerative medicine in the cardiovascular field.

## Background

According to “the top 10 causes of death” announced by the World Health Organization (WHO), heart disease is a leading cause of death in the world. Current therapeutic regimens for heart disease are limited. Heart disease, including heart failure and myocardial infarction, is usually treated with medical therapy, mechanical device implantation, and surgical intervention. When a patient exhibits extremely poor cardiac function, a heart transplant is typically required; however, donor shortage is a major problem for heart transplantation (both in Japan and throughout the world). Thus, cardiac regenerative medicine is an attractive alternative therapy to heart transplantation. For the last two decades, embryonic stem (ES) cells have been used in the field of regenerative medicine due to their self-replication competence and cardiac differentiation ability; however, human ES cells are accompanied by ethical and legal concerns, as well as the threat of immunologic rejection. To solve these problems, Yamanaka and colleagues developed induced pluripotent stem (iPS) cells, which were created by introducing four stem cell-specific transcription factors (Oct3/4, Sox2, c-Myc, and Klf4; collectively, OSKM) into human dermal fibroblasts [1]. However, if iPS cells are to be used in clinical regenerative medicine applications in the future, several issues must be resolved. For example, these cells may demonstrate variable and low cardiomyocyte differentiation efficiency, may require a long time for cardiac maturation, and may show tumorigenicity.

The skeletal muscle master gene, MyoD, was discovered in 1987 and spurred the search for a cardiomyocyte master gene, which has yet to be identified. However, the establishment of iPS cells suggested that cardiac reprogramming could be achieved by concurrent introduction of several transcription factors, rather than a single master gene, into fibroblasts. In fact, we first reported that induced cardiomyocyte-like cells or induced cardiomyocytes (iCMs) could be formed by transducing fibroblasts with genes encoding the cardiac-specific transcription factors, Gata4, Mef2c, and Tbx5 (collectively, GMT) [2]. Prior to our work, an established theory was that the reprogramming and subsequent differentiation of fibroblasts into cardiomyocytes required an iPS cell intermediate; however, our research introduced a new concept in which a direct reprogramming pathway exists for the production of cardiomyocytes from fibroblasts—one that does not involve iPS cells.

Here, we summarize current knowledge about cardiac reprogramming in vitro and in vivo. Furthermore, we discuss future applications of cardiac reprogramming in regenerative medicine.

### Three pathways to generate new cardiomyocytes

**Fig. 1:**
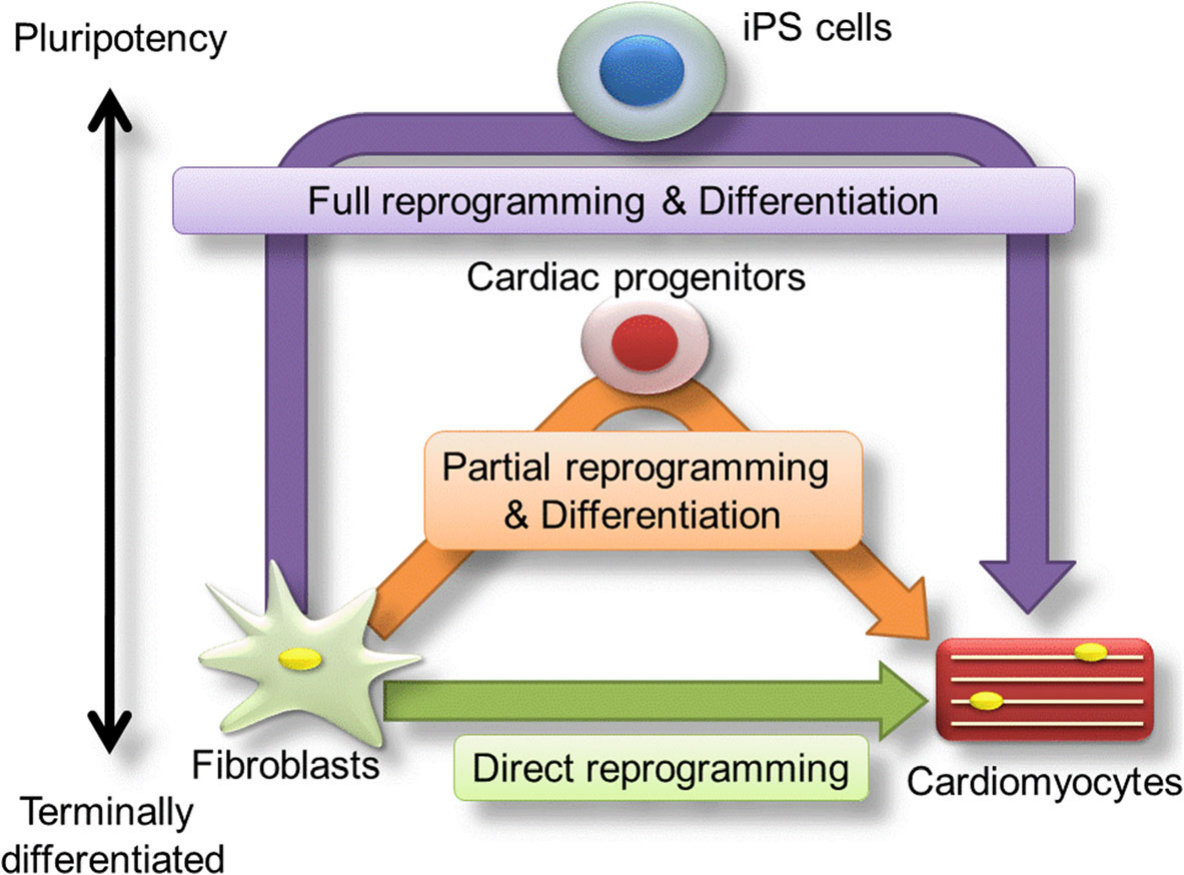
Three major pathways for deriving cardiomyocytes for myocardial regeneration. These strategies include a full reprogramming approach (*purple line*), a partial reprogramming approach (*orange line*), and a direct reprogramming approach (*green line*). Reprogrammed cardiomyocytes can be transplanted into an infarcted or failing heart. Direct injection of transcription factors involved in cardiac reprogramming into the heart may be realized by the direct reprogramming approach, which would not require engrafting of iCMs (derived from reprogrammed fibroblasts) into the heart

The current methods of generating cardiomyocytes from fibroblasts are categorized into three general pathways (see Fig. 1):

1. Full reprogramming of fibroblasts into iPS cells and subsequent cardiac differentiation
2. Partial reprogramming of fibroblasts to cardiac progenitor cells and subsequent differentiation
3. Direct reprogramming of fibroblasts into cardiomyocytes

The cardiomyocytes generated from any of these three pathways can be transplanted into an infarcted or failing heart. The direct reprogramming approach is particularly attractive, as transcription factors involved in cardiac reprogramming can be introduced directly into a heart, bypassing the need for engrafting of iCMs. In this section, we review preclinical and clinical data on these cardiac regeneration strategies and summarize the advantages of each of these three strategies [3].

1. Full reprogramming of fibroblasts into iPS cells and subsequent cardiac differentiation:
  Currently, the major strategy to generate cardiomyocytes requires the full reprogramming of fibroblasts into iPS cells and their subsequent differentiation. This strategy requires complete conversion of fibroblasts to undifferentiated cells (e.g., iPS cells) and differentiation of iPS cells into cardiomyocytes [4].
  Mouse and human iPS cells were established by Takahashi and Yamanaka in 2006 and 2007, respectively [1, 4]. In both instances, iPS cells were derived from fibroblasts by using retroviruses to transduce the fibroblasts with genes encoding four transcription factors (OSKM). iPS cells have brought about a major revolution in regenerative medicine [4]. Because they have a differentiation ability that is similar to ES cells, iPS cells can be exposed to cardiac differentiation protocols that were perfected in ES cells. Following the initial establishment of human iPS cells, functional analyses of iPS cell-derived cardiomyocytes showed that they are embryonic or immature cardiomyocytes rather than adult-type cardiomyocytes [5, 6]. Cardiomyocytes derived from human iPS cells have been used for disease modeling [7], and many laboratories have reported the analysis of models of various diseases using iPS cells from fibroblasts derived from patients or animals with those diseases.
2. Partial reprogramming of fibroblasts to cardiac progenitor cells and subsequent differentiation:
  The second strategy to generate cardiomyocytes requires the generation of partially reprogrammed cells, including cardiac progenitor cells. These cells can be generated during the process of iPS cell generation by exposing fibroblasts to OSKM and can be induced to differentiate into cardiomyocytes. Efe et al. reported an equivalent partial reprogramming method [8]. These researchers reported the successful induction of cardiomyocytes from fibroblast cultures transfected with OSKM, which were subsequently treated with cardiomyocyte-inducing factors.
  If Efe’s method induces partial reprogramming of fibroblasts into cardiac progenitor cells, several cardiomyocytes could be derived from a single fibroblast during this procedure. However, whether this strategy is applicable to human cells remains to be determined.
  Wang et al. demonstrated that Oct4 alone, together with a small-molecule cocktail consisting of SB431542 (transforming growth factor beta (TGFβ) inhibitor), CHIR99021 (glycogen synthase kinase 3 (GSK3) inhibitor), Parnate (lysine-specific demethylase 1 (LSD1)/KDM1 (lysine(K)-specific demethylase1A) inhibitor), and Forskolin (adenylyl cyclase activator) (collectively, SCPF), is sufficient to “erase” the original cell identity, thereby enabling cell conversion with lineage-specific soluble signals [9]. In this case, bone morphogenetic protein (BMP) 4 was added beginning on day 6 after transduction to induce a cardiomyocyte phenotype. By using this strategy, they observed contracting clusters beginning on day 20 and generated 99 ± 17 beating clusters on day 30 after 10,000 mouse embryonic fibroblasts (MEFs) were initially plated [9].
  Very recently, Lalit et al. [10] and Zhang et al. [11] reported two different strategies for reprogramming adult mouse fibroblasts into highly expandable cardiovascular progenitor cells [12]. They induced mouse fibroblasts with combinations of transcription factors and small molecules and succeeded in expanding the cell populations they obtained in chemically defined conditions.
  Lalit et al. [10] found that mouse fibroblasts can be infected with lentivirus harboring a doxycycline-inducible transgene encoding five reprogramming factors (Mesp1, Tbx5, Gata4, Nkx2.5, and Baf60c: collectively, MTGNB), and that self-expanding cardiac progenitor cells can be found with 6-bromoindirubin-30-oxime (BIO; canonical Wnt activator) and leukemia inhibitory factor (LIF; a JAK (Janus kinase) /STAT (signal transducer and activator of transcription) activator). These cells were called induced cardiac progenitor cells and can be expanded over 10^15^-fold and differentiate into cardiomyocytes, endothelial cells, and smooth muscle cells. Transplantation of induced cardiac progenitor cells results in generation of all three of these lineages in vivo and improves survival of mouse after myocardial infarction [10].
  Zhang et al. [11] utilized secondary MEFs, which transiently overexpress the four Yamanaka factors (OSKM) and showed that Yamanaka factor expression plus the JAK inhibitor JI1 and BACS (BMP4, activin A (the member of the transforming growth factor beta (TGF-β)), CHIR99021, and SU5402 (fibroblast growth factor receptor (FGFR)-specific tyrosine kinase inhibitor)) reprograms mouse fibroblasts into cardiac progenitor cells with a high capacity for expansion. These cells were named induced expandable cardiac progenitor cells, and they differentiate into cardiomyocytes, endothelial cells, and smooth muscle cells in vitro and after transplantation into myocardial infarcted hearts [11].
3. Direct reprogramming of fibroblasts into cardiomyocytes:
  Recently, a third strategy was developed as a new method to directly convert fibroblasts into another cell type by introducing single or multiple transcription factors. In 2010, Vierbuchen et al. succeeded in generating neuronal-like or induced neuronal cells by introducing three genes encoding transcription factors (Ascl1, Brn2, and Mytl1) necessary for neuronal differentiation into mouse fibroblasts [13]. This was the first successful report of direct reprogramming of fibroblasts into a specific cell type (without an iPS cell step) using organ-specific transcription factors.
  Following the work of Vierbuchen and colleagues, we reported that neonatal mouse cardiac fibroblasts could be converted into cardiomyocyte-like cells or iCMs following introduction of genes encoding cardiac-specific transcription factors (Gata4, Mef2c, Tbx5: GMT) [2]. More recently, Sekiya et al. reported the direct reprogramming of hepatocyte-like cells or induced hepatocytes from mouse fibroblasts [14]. Direct reprogramming technology converts terminally differentiated fibroblasts into another organ cell type and does not require the formation of iPS cells. In time, this strategy may provide a safe and novel alternative to heart transplants. We summarize the three strategies used to derive cardiomyocytes from fibroblasts in Table 1.

**Table 1:** Three strategies to generate cardiomyocytes from fibroblasts. The properties of the cells, advantages, and disadvantages of the strategies are shown

Strategy	Full reprogramming via iPS cells	Partial reprogramming via cardiac progenitor cells (CPCs)	Direct cardiac reprogramming
Cell state	iPS cells (pluripotent)	Cardiac progenitor cells (multipotent)	Differentiated cardiomyocytes (unipotent)
Properties	1. Pluripotent cells 2. Bypass ethical and legal problems (compared to ES cells) 3. Not accompanied by the problem of immunologic refusal	Multipotent CPCs can generate vascular and cardiac cells	Transdifferentiation without an undifferentiated (intermediate) state (i.e., iPS cells, CPCs)
Advantages	Engraftment of ES cell-derived cardiomyocytes is possible in large animal models, accompanied by improved heart function	A short culture period (weeks) required to produce cardiomyocytes, compared with iPSC-mediated cardiomyocytes	1. In vivo reprogramming 2. Takes 4 weeks to generate functional cardiomyocytes 3. Lack of tumor formation 4. Generating only cardiomyocytes
Disadvantages	1. Risk of teratoma formation 2. A long culture period (months) is required to generate cardiomyocytes 3. Immaturity of stem cell-derived cardiomyocytes	1. Uncertain mechanism of OSKM-mediated CPC induction 2. Risk of tumor formation	1. Immaturity of iCMs 2. Low efficiency of full reprogramming into functional cardiomyocytes 3. iCMs are not proliferative

### Direct cardiac reprogramming in vitro

#### Generation of mouse iCMs

Five years ago, we discovered that neonatal cardiac fibroblasts could be reprogrammed directly to form iCMs, without going through an intermediate iPS cell phase (see Table 2). Since then, multiple laboratories have reported the generation of iCMs using various methods. As cell sources for the generation of iCMs, we and others used cardiac fibroblasts, tail-tip fibroblasts, or MEFs derived from reporter mice that express a fluorescent protein when a cardiac-specific promoter, α-myosin heavy chain or cardiac troponin T (cTnT), is activated. To overexpress reprogramming factors in fibroblasts, researchers have used one of two techniques: (1) genes encoding cardiac-specific transcription factors (Gata4, Mef2c, Tbx5, Hand2, Myocd, etc.) were introduced into cells with viral vectors (retroviruses, lentiviruses, adenoviruses, etc.); or (2) the lipofection method was used to transfect cells with cardiac-specific microRNAs (miRs). The reprogramming efficiency can be quantified by counting the number of cells that express the cardiac reporter or protein (by flow cytometry or fluorescence-activated cell sorting) 1–3 weeks after introduction of reprogramming factors into fibroblasts. As part of the functional analysis, these cells were further evaluated for spontaneous beating, calcium homeostasis, and action potentials. Based on our epoch-making study, Song et al. were able to produce functional iCMs (identified as cTnT(+) cells) from adult cardiac fibroblasts and tail-tip fibroblasts by adding a gene encoding a fourth transcription factor—Hand2—to GMT (collectively GHMT) [15]. However, Chen et al. showed the difficulty in generating functional cardiomyocytes through induction with GMT and emphasized the need to examine the reprogramming mechanisms and epigenetic changes induced with this transcription factor cocktail [16].

**Table 2:** Direct/partial reprogramming of mouse/rat fibroblasts to cardiomyocytes in vitro

Reprogramming factors	Supplement agents	Species	Starting cell source	Efficiency	Comments	References
Gata4, Mef2c, Tbx5 (GMT)		Mouse	Adult cardiac fibroblasts (CFs) and tail-tip fibroblasts (TTFs)	20~30 % cTnT+ cells after 1 week	Beating after 4 weeks (CFs) Using retrovirus and lentivirus	[2]
Oct4, Sox2, Klf4 (, c-Myc)	JAK inhibitor I, BMP4	Mouse	Mouse embryonic fibroblasts (MEFs)	40 % cTnT(+) after 18 days	Partial reprogramming Beating after 18 days in cluster Using secondary MEFs harboring doxycycline-inducible transgenes encoding reprogramming factors.	[8]
Oct4	ALK4/5/7 inhibitor, GSK3 inhibitor, LSD/KDM1 inhibitor, BMP4	Mouse	MEFs, TTTFs	Make clusters	Partial reprogramming Beating after 20 days Without any evidence of entrance into the pluripotent state Using doxycycline inducible-expression lentivirus-based gene delivery system	[9]
Mesp1, Tbx5, Gata4, Nkx2.5, Baf60c (MTGNB)	BIO, LIF	Mouse	MEFs, CFs	Passage over 20 times, expand more than 10^15^-fold	Partial reprogramming Using a doxycycline-inducible lentivirus vector	[10]
Oct4, Sox2, Klf4, c-Myc (OSKM)	JAK inhibitor (JI1), BACS (BMP4, Activin A,CHIR99021, and SU5402)	Mouse	MEFs, TTFs	Expanded more than 10^10^-fold	Partial reprogramming Using secondary MEFs harboring doxycycline-inducible transgenes encoding reprogramming factors	[11]
Gata4, Mef2c, Tbx5 (GMT)		Mouse	CFs and TTFs	35 % cTnT+ cells	Beating after 4~5 weeks Using retrovirus	[15]
Gata4, Mef2c, Tbx5 (GMT)		Mouse	CFs and TTFs	35 % cTnT+ cells	Beating after 4~5 weeks Using lentivirus	[16]
Myocd, Mef2, Tbx5 (3F-Myocd)		Mouse	MEFs and neonatal CFs	2.5 % αMHC+ cells	Analysis of ion-channel Using lentivirus	[17]
miR-1, miR-133, miR-208, miR-499	JAK inhibitor 1	Mouse	CFs	13~28 % αMHC+ cells	Added JAK inhibitor Only micro RNAs	[18]
GMT, Hand2, Nkx2-5 (HNGMT)		Mouse	MEFs, CFs	Almost 5 % the calcium indicator GCaMP(+) cells	Using the induction of calcium oscillation for screening Utilizing an inducible-expression lentivirus-based gene delivery system (lentivirus)	[19]
GMT, Mesp1, Myocd, Smarcd3 (Baf60c), SRF		Mouse	MEFs	2.4 % αMHC+ cells	Using lentivirus	[20]
GMT, miR-133		Mouse	MEFs, CFs	40~50 % αMHC+ cells (MEFs)	Beating after 10 days Using retrovirus	[21]
Gata4, Mef2c-MyoD fusion, Tbx5, Hand2		Mouse	MEFs, TTFs	10–20 % cTnT(+) cells	Beating after 7 days Using retrovirus	[22]
Mef2c-P2A-Gata4-T2A-Tbx5		Mouse	Adult CFs		Tenfold increase in beating iCMs Using polycistronic vectors (retrovirus)	[23]
Gata4, Mef2c, Tbx5, Hand2, Nkx2-5 (HNGMT)	TGFβ inhibitor	Mouse	MEFs, CFs	17 % the calcium indicator GCaMP(+) cells	Beating approximately fivefold compared to 16) Using a doxycycline-inducible lentivirus vector	[24]
Gata4, Mef2c, Tbx5, Hand2 (GHMT)	Akt1	Mouse	MEFs, CFs, TTFs	50 % of reprogrammed MEFs beating	Beating after 3 weeks Using retrovirus	[25]
(−)	CHIR99021, RepSox, Forskolin, VPA, Parnate, TTNPB, DZnep	Mouse	MEFs, TTFs		Chemical reprogramming Beating after 20–24 days	[26]
Gata4, Mef2c, Tbx5, Hand2 (GHMT), miR-1, miR-133	A83-01 (inhibitor of TGF-β1), Y-27632 (inhibitor of ROCK)	Mouse	MEFs, CFs	60 % cTnT(+) cells after	Beating 2 weeks Using retrovirus	[27]
Gata4, Mef2c, Tbx5 (Hand2)	FGF2, FGF10, VEGF, IWR-1	Mouse	MEFs, CFs, TTFs	10–20 % αMHC(+) cells after 1 week	1 % Beating after 4 weeks (GMT) 5~9 % Beating after 4 weeks (GHMT) Using retrovirus	[28]
Mef2c, Tbx5	FGF2, FGF10, VEGF	Mouse	MEFs	3 % αMHC(+) cells after 1 week	Beating after 4 weeks Using retrovirus	[28]

Protze et al. introduced 120 combinations of factors into MEFs using a pool of 10 transcription factors in an attempt to induce cardiac differentiation and confirmed cardiomyocyte properties in treated cells through gene expression analyses. They showed that the 3F-Myocd combination (Mef2c, Tbx5, and Myocd, in which Myocd was substituted for Gata4) may result in cardiomyocytes that are more differentiated than with other combinations [17].

In addition, Jayawardena et al. introduced only the microRNAs, miR-1, miR-133, miR-208, and miR-499, into neonatal cardiac fibroblasts and succeeded in generating iCMs, distinguishing this report from other research. As microRNAs are not incorporated into host chromosomes during transient expression, microRNA-mediated induction may be safer for human applications [18]. This research also suggested that culture conditions are vital to cardiomyocyte induction, as expression of α-myosin heavy chain-cyan fluorescence protein (CFP) in transgenic mice increased nearly tenfold when a JAK inhibitor was added to the culture medium.

Addis et al. reported the benefits of adding Nkx2-5 and Hand2 to GMT if both factors were added to GMT. Using a transgenic calcium fluorescent reporter driven by a cardiomyocyte-specific gene promoter, they demonstrated that infection with GMT, Hand2, and Nkx2-5 (collectively HNGMT) results in the most efficient generation of functional cardiomyocytes [19]. Christoforou et al. determined that overexpression of Myocd and Srf (serum response factor) transcription factors, alone or in conjunction with Mesp1 and Smardcd3 (Baf60c), enhances the basal cardiac-inducing effects of GMT. Through global gene expression analysis, they demonstrated the significantly greater cardiac-inducing effects of Myocd and Srf compared to GMT alone [20].

In 2014, we demonstrated that miR-133 overexpression paired with GMT generates sevenfold more beating iCMs from MEFs compared to GMT treatment alone; this treatment also shortened the duration required to induce beating iCMs (from 30 to 10 days). Furthermore, we found that miR-133-mediated Snai1 repression is critical for cardiac reprogramming in adult mouse (and human cardiac) fibroblasts, and that silencing fibroblast signatures via miR-133/Snai1 is a key molecular roadblock during cardiac reprogramming [21]. Importantly, this was the first study to demonstrate a molecular mechanism underlying cardiac reprogramming by defined factors.

Hirai et al. fused a transactivation domain from MyoD to individual factors in the GHMT cocktail and found that fusion of the Mef2c C-terminus with the MyoD transactivation domain plus wild-type Gata4, Hand2, and Tbx5 accelerates cardiac reprogramming and generates larger beating clusters from MEFs with a 15-fold greater efficiency than GHMT without the fusion [22]. This result is consistent with the observation that reprogramming requires high levels of gene expression and activity to overcome the high barrier of cellular stability that is inherently present in adult somatic cells.

Wang et al. generated six polycistronic constructs to include all ordered combinations of Gata4, Mef2c, and Tbx5 with identical self-cleaving 2A sequences and showed distinct protein levels of the three transcription factors based on the splicing order [23]. They further demonstrated that relatively higher protein levels of Mef2c with modest levels of Gata4 and Tbx5 lead to more efficient cardiac reprogramming, and an optimized MGT combination with puromycin selection results in an over tenfold increase in beating iCMs. This report convincingly showed that the protein ratio of cardiac reprogramming factors could greatly influence the efficiency and quality of iCMs.

#### Small molecules promote the reprogramming of mouse iCMs

Recently, multiple groups have shown that modification of reprogramming factors can promote cardiac reprogramming. In particular, by stimulating or inhibiting the signaling pathways involved in generation of cardiomyocytes, they could improve cardiac reprogramming efficiency. Cardiac reprogramming can also be affected by cell culture conditions. These recent findings provide new insights into the molecular mechanisms underlying cardiac conversion of fibroblasts and will enhance efforts to generate cardiomyocytes for clinical applications (see Table 2).

Ifkovits et al. visualized the induction of calcium oscillations in reprogrammed cells with a transgenic calcium reporter, GCaMP5 (Ca^2+^ probe composed of a single GFP 5), driven by a cardiac-specific gene promoter. They found that a combination of five cardiac transcription factors, GMT, Hand2, and Nkx2.5 (GMTHN), more efficiently reprograms MEFs. They also found that GCaMP5 helps track the location of rare beating iCMs that represent fully reprogrammed cells. With the same method, they found that a small molecule inhibitor of TGF-β, SB431542, increases reprogramming efficiency via GMTHN up to nearly fivefold and generates more beating iCMs from MEFs [24].

Zhou et al. discovered that Akt/protein kinase B dramatically improves the efficiency of reprogramming fibroblasts to iCMs by the cardiac transcription factors GHMT. Approximately 50 % of reprogrammed MEFs displayed spontaneous beating after 3 weeks of induction by Akt plus GHMT. Insulin-like growth factor 1 and phosphoinositol 3-kinase act upstream of Akt, whereas the mitochondrial target of rapamycin complex 1 and forkhead box O3 act downstream of Akt to influence fibroblast-to-cardiomyocyte reprogramming [25].

Fu et al. reported generation of automatically beating cardiomyocyte-like cells from mouse fibroblasts using only chemical cocktails (CHIR99021, RepSox (inhibitor of the TGFβ receptor-1/ALK5), Forskolin, VPA (valproic acid; histone deacetylase inhibitor), Parnate, TTNPB (Arotinoid acid; a synthetic stilbene analog of retinoic acid (RA)), DZnep (3-Deazaneplanocin A hydrochloride; histone methyltransferase EZH2 (enhancer of zeste homolog 2) inhibitor)) [26]. These chemically induced cardiomyocyte-like cells express cardiomyocyte-specific markers and possess typical cardiac calcium transients and electrophysiological features [26].

Zhao et al. reported that inhibition of the TGF-β1 or Rho-associated kinase (ROCK) pathways converts embryonic fibroblasts into functional cardiomyocyte-like cells by forced expression of GMT or GHMT, with an efficiency of up to 60 %. Furthermore, inhibition of TGF-β1 or ROCK signaling dramatically enhances full reprogramming, with spontaneously beating cardiomyocytes emerging in less than 2 weeks with GHMT alone [27].

In 2015, we demonstrated that a combination of fibroblast growth factor (FGF) 2, FGF10, and vascular endothelial growth factor (VEGF) promotes cardiac reprogramming in defined serum-free conditions, increasing spontaneously beating iCMs by 100-fold compared with other conventional serum-based conditions. Mechanistically, FGF2, FGF10, and VEGF activate multiple cardiac transcriptional regulators and convert partially reprogrammed cells into functional iCMs through the p38 mitogen-activated protein kinase and phosphoinositol 3-kinase/AKT pathways. Moreover, our cocktail enables cardiac reprogramming with only Mef2c and Tbx5 [28].

#### Generation of human iCMs

Three studies including ours applied the concept of direct reprogramming to neonatal and adult human fibroblasts in 2013 [29–31] (see Table 3). Nam et al. reported that a combination of genes encoding four transcription factors (Gata4, Hand2, Tbx5, and Myocd) and two muscle-specific microRNAs (miR-1 and miR-133) can reprogram up to 20 % of human fibroblasts into cTnT(+) cells (presumptive cardiomyocytes). Furthermore, a subset of iCMs derived from human cardiac fibroblasts demonstrated spontaneous beating after 11 weeks in culture [29]. Similarly, Fu et al. reported that a mixture of genes encoding seven transcription factors (Gata4, Mef2c, Tbx5, Mesp1, Myocd, Zfpm2, Esrrg) can induce human cardiomyocyte gene expression in treated fibroblasts [30]. This work also demonstrated that this mixture of reprogramming factors generates epigenetically stable human iCMs, and that TGF-β signaling improves the efficiency of human iCM reprogramming [30]. Finally, we found that a combination of genes encoding five transcription factors (Gata4, Mef2c, Tbx5, Mesp1, and Myocd) can reprogram human fibroblasts into beating, cardiomyocyte-like cells with action potentials when co-cultured with rat cardiomyocytes [31]. Islas et al. used two transcription factors (Mesp1 and Ets-2) in activin A- and BMP2-treated cells to reprogram human dermal fibroblasts into cardiac progenitor-like cells, which could then differentiate into cardiomyocyte-like cells [32]. Despite these promising results, direct cardiac reprogramming is less efficient in human cells compared to mouse fibroblasts.

**Table 3:** Direct reprogramming of human fibroblasts to cardiomyocytes in vitro

Reprogramming factors	Supplement agents	Species	Starting cell type	Efficiency	Comments	References
Gata4, Hand2, Myocd, Tbx5, miR-1, miR-133,		Human	Human neonatal foreskin fibroblasts (HFF), adult human cardiac fibroblasts (AHCFs) and and adult human dermal fibroblasts (AHDFs)	~20 % cTnT(+) cells (HFFs) 13 % cTnT(+) cells (AHCFs) 9.5 % cTnT(+) cells (AHDFs)	Bating after 11 weeks (AHCFs) Using retrovirus	[29]
Gata4, Mef2c, Tbx5, Mesp1, Myocd, Zfp42/Rex1, ESRRG		Human	Human cardiac fibroblasts (HCF)	35 % cTnT(+) cells	Using retrovirus and lentivirus	[30]
Gata4, Mef2c, Tbx5, Mesp1, Myocd		Human	Adult cardiac human fibroblasts (AHCFs) and adult dermal fibroblasts (AHDFs)	5 % αactinin(+) and cTnT(+) cells	Beating co-cultured with mouse cardiomyocytes Using retrovirus	[31]
Ets2, Mesp1		Human	Adult dermal human fibroblasts (AHDFs)	2.3 % αMHC(+) cells	Cells expressing the cardiac mesoderm marker KDR(+) using lentivirus	[32]
Gata4, Mef2c, Tbx5, Mesp1, Myocd, miR-133		Human	Human cardiac fibroblasts (HCFs)	23~27 % cTNT(+) cells	Using retrovirus	[21]
Gata4, Mef2c, Tbx5, Hand2	Add supplement agents (BMP4, activin A, FGF2, IWR1)	Human	Adult human dermal human fibroblasts (AHDFs)		Cardiac progenitor cells (?) Using retrovirus	[33]
(−)	9 compounds (9C; CHIR99021, A83-01, BIX01294, SC1, Y27632, OAC2, SU16F, and JNJ10198409)	Human	Human foreskin fibroblast (HFF) Human fetal lung fibroblasts (HLFs)	6.6 ± 0.4 % of cTNT(+) cells	Chemical reprogramming	[34]

Muraoka et al. induced 2–8 % of α-actin(+)/cTnT(+) cells with lentiviral transduction of Gata4, Mef2c, Tbx5, Mesp1, and Myocd into human cardiac fibroblasts (HCFs). Interestingly, by adding miR-133 to the reprogramming cocktail, they increased the efficiency of iCM generation to 23–27 % [21].

In 2015, Li et al. reported that the combination of QQ-reagent-modified Gata4, Hand2, Mef2c, and Tbx5 and sevral cytokines (BMP4, activin A, FGF2, IWR1 (Wnt pathway inhibitor)) reprogrammed human dermal fibroblasts (HDFs) into CPCs [33]. Like what Yamamakawa et al. pointed out [28], the protein-transduction method can directrely program with high efficiency. And finally, Cao et al. demonstrated that cardiomyocyte-like cells can be generated by treating human fibroblasts with a combination of nine compounds (CHIR99021, A83-01 (Inhibitor of TGF-beta type I receptor), BIX01294 (a histone methyltransferase (HMTase) inhibitor), SC1 (ERK 1 inhibitor), Y27632 (ROCK inhibitor), OAC2 (Oct4-activating compound 2), SU16F (inhibitor of platelet-derived growth factor receptor-beta (PDGFR beta), and JNJ10198409 (inhibitor of platelet-derived growth factor receptor tyrosine kinase (PDGF-RTK))). The chemically induced cardiomyocyte-like cells uniformly contracted and resembled human cardiomyocytes in their transcriptome, epigenetic, and electrophysiological properties [34].

 These protein reprogramming strategies have the promising approaches for future regenerative medicine both in vitro and in vivo. But the conversion of fibroblasts into human iCMs is not easy, compared with mouse iCMs. Therefore, further research is essential to identify optimal reprogramming factors (transcription factors, microRNAs, etc.) as well as culture conditions (small molecules, cytokines, etc.) for improving reprogramming efficiency and use in clinical applications [33, 34].

### Direct cardiac reprogramming in vivo

The most exciting potential for cardiac transcription factor-based reprogramming is the possibility of using this technology in vivo. Injection of reprogramming factors directly into the damaged heart may convert endogenous cardiac fibroblasts, which represent >50 % of all cardiac cells, into new functional cardiomyocytes. This in vivo reprogramming approach may have several advantages over cell transplantation-based therapy. First, the process is simple. Second, avoiding the induction of pluripotent cells before cardiac differentiation would greatly lower the risk of tumor formation. Third, direct injection of defined factors obviates the need for cell transplantation, for which long-term cell survival remains challenging [35–37] (see Table 4).

**Table 4:** Direct reprogramming of fibroblasts to cardiomyocytes in vivo

Reprogramming factors	Gene transduction	Species	Disease model	Comments	References
Gata4, Mef2c, Tbx5 (GMT)	Retrovirus	Mouse	Myocardial infarction (MI)	Injected reprogrammed cells	[2]
Mesp1, Tbx5, Gata4, Nkx2.5, Baf60c (MTGNB)	Using a doxycycline-inducible lentivirus vector	Mouse	MI	Improved survival after myocardial infarction (MI) Differentiate into cardiomyocytes, smooth muscle cells, and endothelial cells	[10]
Oct4, Sox2, Klf4, c-Myc (OSKM)	(−) Using secondary MEFs harboring doxycycline-inducible transgenes encoding reprogramming factors	Mouse	MI	Improves heart function after MI Reduce infract area Differentiate into cardiomyocytes, smooth muscle cells, and endothelial cells	[11]
Gata4, Hand2, Mef2c, Tbx5 (GHMT)	Retrovirus	Mouse	MI	Injected virus 2.4~6.5 % (border zone) reduction of 50 % in scar zone	[15]
Gata4, Mef2c, Tbx5 (GMT)	Lentivirus	Mouse	MI	Injected reprogrammed cells	[16]
Gata4, Mef2c, Tbx5 (GMT)	Retrovirus	Mouse	MI	Injected virus Almost 35 % of iCMs in the border/infarct area Improvement 10 % in ejection fraction	[38]
Gata4, Mef2c, Tbx5 (GMT) 3F-2A system	Polycistronic vectors (retrovirus)	Mouse	MI	Injected virus 1~3 % (border zone)	[31]
miR-1, miR-133, miR-208, miR-499	microRNA	Mouse	MI	Added JAK inhibitor ~1 % (border zone)	[18]
GMT and Vegf (GMT/VEGF)	Lentivirus	Rat	MI	Injected virus Improvement in ejection fraction fourfold greater in GMT/VEGF vs GMT only	[40]

For example, cardiac fibroblasts in an infarcted area of a heart could be targeted for cardiogenic reprogramming, resulting in the formation of new cardiomyocytes in situ. In 2012, multiple groups including us demonstrated the transdifferentiation of fibroblasts into cardiomyocytes in vivo. Olson’s and Srivastava’s groups used the Cre recombinase driven by fibroblast-specific promoters to trace the cell fate of cardiac fibroblasts and subsequent cardiomyocyte transdifferentiation.

Qian et al. used the periostin and fibroblast-specific protein 1 (FSP-1) promoter Cre transgenic mice and found that fibroblasts in infarcted hearts are converted into cardiomyocyte-like cells by GMT retroviral gene transfer; global function also restored in treated hearts [38]. Following direct injection of GMT retroviruses into infarcted mouse hearts, this work demonstrated that almost 35 % of cardiomyocytes in the infarcted area or its border were newly generated iCMs derived from resident cardiac fibroblasts. Furthermore, half of these iCMs showed well-organized sarcomeric structures and exhibited functional characteristics of adult ventricular cardiomyocytes, including cellular contraction, electrophysiological properties, and functional coupling to other cardiac cells. These observations suggested that in vivo reprogramming generates functional iCMs more efficiently than in vitro reprogramming [38]. In contrast to the work of Qian et al., Song et al. added Hand2 to the GMT cocktail (creating a GHMT cocktail) and utilized FSP-1 promoter Cre transgenic and Tcf21-iCre knock-in mice for fibroblast lineage tracing. They reported that GHMT retroviral injection into mouse infarcted hearts converts endogenous cardiac fibroblasts into functional cardiomyocyte-like cells in vivo [15]. These researchers also demonstrated that approximately 6 % of cardiomyocytes in the infarcted area or its border were newly generated cardiomyocyte-like cells with clear striations and functional properties similar to those of endogenous ventricular cardiomyocytes. Twelve weeks after myocardial infarction, Song et al. also demonstrated that the scar zone of infarcted hearts was reduced by 50 %, and the ejection fraction was increased twofold in GHMT-treated mice compared to controls [15].

We generated a polycistronic retrovirus expressing GMT. This polycistronic retrovirus, which expresses GMT at near equimolar levels from the same promoter, was generated using self-cleaving 2A peptides [39]. We co-injected polycistronic GMT (3F2A) and reporter genes (e.g., GFP) to determine cardiac induction from non-myocytes. We found that gene transfer of this polycistronic GMT retrovirus induces more mature cardiomyocyte-like cells (as evidenced by sarcomeric structures) than those generated by the injection of three separate vectors.

Mathison et al. injected a mixture of GMT retroviruses and VEGF into infarcted myocardium areas in rats. Infarcted areas were reduced in rats treated with VEGF compared to those only treated with GMT. This reduction in the scar in the infarcted area may be due to VEGF-mediated neovascularization or some other unknown mechanisms [40].

Direct injection of lentiviruses containing four microRNAs (miR-1, miR-133, miR-208, and miR-499) into mouse infarcted hearts converts resident cardiac fibroblasts into cardiomyocyte-like cells in vivo. After injection of these microRNAs, Jayawardena et al. reported that approximately 1 % of the infarcted area contained new iCMs; however, this work did not report on whether ejection fraction improved after microRNA injection [18].

For clinical applications, the development of a non-viral delivery method, including chemically synthesized molecules and microRNAs, may be a very attractive therapeutic approach, because non-viral factors do not integrate into the host chromosomes. Of note, these results suggest that the abundant pool of endogenous cardiac fibroblasts could be a cell source for new cardiomyocytes via direct reprogramming and that this new technology may improve cardiac function and reduce scar size after myocardial infarction. These studies clearly demonstrate that iCMs reprogrammed in vivo are more mature than those reprogrammed in vitro, suggesting that the effects of the in vivo environment, such as mechanical stretch, local signals, and the extracellular matrix, enhance the quality of iCMs in the native heart.

## Conclusions

We reviewed the three different reprogramming strategies that are being developed in the field of cardiac regenerative medicine. Although all strategies (iPS cell approach, partial reprogramming, and direct reprogramming) have been utilized by many researchers, these strategies each have several problems that must be overcome prior to clinical application [41, 42].

The heart is composed of various groups of cells, including blood vessel endothelial cells, smooth muscle cells, nerve cells, and cardiac fibroblasts. Judging from the absolute number of cells comprising the heart, cardiomyocytes only account for approximately 30 % of heart cells, whereas cardiac fibroblasts constitute approximately 50 % of this organ. When a large number of cardiomyocytes die due to necrosis caused by myocardial infarction, the number of cardiac fibroblasts increases in the infarcted area. Heart rupture can be prevented by replacing an infarcted area with fibrous tissue; however, fibroblasts can result in low cardiac function and a potentially fatal arrhythmic focus. Direct reprogramming technology may provide an ideal treatment that could bypass the formation of cardiac fibroblasts in an infarcted region, instead resulting in new cardiomyocyte formation if certain genes are efficiently introduced into cardiac tissue [43].

Today, almost all reports of successful direct cardiac reprogramming have been generated with retroviruses or lentiviruses (Tables 2, 3, and 4). These reports involve integration in the host cell genome with an identified risk for insertional mutagenesis. To circumvent such risks which are deemed incompatible with therapeutic prospects, significant progress has been made with transgene-free reprogramming methods based on other kinds of virus, microRNA [15], or the cocktail of small molecules [26, 34] to achieve conversion into cardiomyocytes.

In the future, many scientists will examine the feasibility of a novel reprogramming process based on transgene-free methods using adenovirus, microRNAs, non-viral episomal expression vectors, and protein transduction.

However, for direct reprogramming to be used in clinical applications, the cardiac reprogramming efficiency induced by this method must be optimized. The generation of sufficient numbers of fully reprogrammed cells in vitro will also be valuable for drug toxicity studies and drug screening. Currently, the reprogramming efficiency of fibroblasts into mature cardiomyocytes is variable and low. Although several reports have described direct reprogramming of human cardiac fibroblasts into cardiomyocytes, further study is required for optimization.

On the other hand, current iCM technology is quite efficient for in vivo reprogramming, and the iCM in vivo reprogramming approach has several advantages over cell-based transplantation therapy. Because reprogramming factors are directly injected into the heart, no issues arise concerning the homing, survival, or migration of transplanted cells.

Future identification of small molecules or secreted proteins that could replace each transcription factor, as has been performed for iPS cell reprogramming, may allow an alternative to gene therapy. We hope to utilize regenerative medicine-based therapies to treat patients with severe heart failure, potentially employing cardiac muscle cells derived from iPS cells and iCMs.

## Abbreviations

A83-01: Inhibitor of TGF-β type I receptor, ALK5 kinase
ADHF: Adult human dermal fibroblasts
AHCF: Adult human cardiac fibroblasts
ALK: Activin receptor-like kinase
AS8351: 2-Hydroxy-1-naphthylaldehyde isonicotinoyl hydrazine, histone demethylase inhibitor
BIO: 6-Bromoindirubin-30-oxime, canonical Wnt activator
BIX01294: (2-(Hexahydro-4-methyl-1H-1,4-diazepin-1-yl)-6,7-dimethoxy-N-[1-(phenylmethyl)-4-piperidinyl]-4-quinazolinamine trihydrochloride), a histone methyltransferase (HMTase) inhibitor)
BMP: Bone morphogenetic protein
CF: Cardiac fibroblast
CFP: Cyan fluorescence protein
CHIR99021: 6-{2-[4-(2,4-Dichloro-phenyl)-5-(5-methyl-1H-imidazol-2-yl)-pyrimidin-2-ylamino]-ethylamino}-nicotinonitrile), GSK3 inhibitor
cTnT: Cardiac troponin T
DZnep: 3-Deazaneplanocin A hydrochloride; histone methyltransferase (EZH2 inhibitor)
ERK: Extracellular signal-regulated kinase
ES cells: Embryonic stem cells
EZH2: Enhancer of zeste homolog 2
FGF: Fibroblast growth factor
FGFR: Fibroblast growth factor receptor
FSP-1: Periostin and fibroblast-specific protein 1
GCaMP: Ca2+ probe composed of a single GFP
GMT: Gata4, Mef2c, and Tbx5
GSK3: Glycogen synthase kinase 3
HCF: Human cardiac fibroblasts
HFF: Human neonatal foreskin fibroblasts
HLF: Human fetal lung fibroblasts
HMTase: Methyltransferase inhibitor
iCMs: Induced cardiomyocytes
iPS cells: Induced pluripotent stem cells
IWR1: 4-[(3aR,4S,7R,7aS)-1,3,3a,4,7,7a-hexahydro-1,3-dioxo-4,7-methano-2H-isoindol-2-yl]-N-8-quinolinyl-benzamide, Wnt pathway inhibitor
JAK: Janus kinase
JI1: JAK inhibitor 1
JNJ10198409: *N*-(3-Fluorophenyl)-2,4-dihydro-6,7-dimethoxyindeno[1,2-c]pyrazol-3-amine, inhibitor of platelet-derived growth factor receptor tyrosine kinase (PDGF-RTK)
KDM1: Lysine(K)-specific demethylase1A
LIF: Leukemia inhibitory factor, a JAK/STAT activator
LSD1: Lysine-specific demethylase 1
MEF: Mouse embryonic fibroblast
miR: microRNA
OAC2: *N*-1H-indol-5-yl-benzamide, Oct4-activating compound 2
OKSM: Oct3/4, Sox2, c-Myc, and Klf4
Parnate: Tranylcypromine, LSD1/KDM1 inhibitor)
RepSox: E-616452, 2-(3-(6-Methylpyridine-2-yl)-1H-pyrazol-4-yl)-1,5-naphthyridine
ROCK: Rho-associated kinase
SB431542: 4-[4-(1,3-Benzodioxol-5-yl)-5-(pyridin-2-yl)-1H-imidazol-2-yl]benzamide, TGFβ inhibotor
SB431542: 4-[4-(1,3-Benzodioxol-5-yl)-5-(2-pyridinyl)-1H-imidazol-2-yl]-benzamide, ALK4/5/7 inhibitor
SC1: *N*-(3-(7-(1,3-dimethyl-1H-pyrazol-5-ylamino)-1-methyl-2-oxo-1,2-dihydropyrimido[4,5-d]pyrimidin-3(4H)-yl)-4-methylphenyl)-3-(trifluoromethyl)benzamide, Pluripotin, ERK 1 inhibitor
Srf: Serum response factor
STAT: Signal transducer and activator of transcription
SU16F: 5-[1,2-Dihydro-2-oxo-6-phenyl-3H-indol-3-ylidene)methyl]-2,4-dimethyl-1H-pyrrole-3-propanoic acid, inhibitor of platelet-derived growth factor receptor-beta (PDGFRβ)
SU5402: 3-[3-(2-Carboxyethyl)-4-methylpyrrol-2-methylidenyl]-2-indolinone, fibroblast growth factor receptor (FGFR)-specific tyrosine kinase inhibitor
TGF-β: Transforming growth factor beta
TTF: tail tip fibroblast
TTNPB: 4-[(E)-2-(5,6,7,8-Tetrahydro-5,5,8,8-tetramethyl-2-naphthalenyl)-1-propenyl]benzoic acid (Arotinoid acid; a synthetic stilbene analog of retinoic acid (RA))
VEGF: Vascular endothelial growth factor
VPA: Valproic acid
WHO: the World Health Organization
Y-27632: (trans-4-[(1R)-1-Aminoethyl]-*N*-4-pyridinylcyclohexanecarboxamide), inhibitor of ROCK

## References

[1] Takahashi K, Tanabe K, Ohnuki M (2007). Induction of pluripotent stem cells from adult human fibroblasts by defined factors. Cell.

[2] Ieda M, Fu JD, Delgado-Olguin P (2010). Direct reprogramming of fibroblasts into functional cardiomyocytes by defined factors. Cell.

[3] David R, Franz WM (2011). From pluripotency to distinct cardiomyocytes subtypes. Physiology.

[4] Takahashi K, Yamanaka S (2006). Induction of pluripotent stem cells from mouse embryonic and adult fibroblast cultures by defined factors. Cell.

[5] Narazaki G, Uosaki H, Teranishi M (2008). Directed and systematic differentiation of cardiovascular cells from mouse induced pluripotent stem cells. Circulation.

[6] Zhang J, Wilson GF, Soerens AG (2009). Functional cardiomyocytes derived from human induced pluripotent stem cells. Circ Res.

[7] Moretti A, Bellin M, Welling A (2010). Patient-specific induced pluripotent stem-cell models for long QT-syndrome. N Engl J Med.

[8] Efe JA, Hilcove S, Kim J (2011). Conversion of mouse fibroblasts into cardiomyocytes using a direct reprogramming strategy. Nat Cell Biol.

[9] Wang H, Cao N, Spencer CI (2014). Small molecules enable cardiac reprogramming of mouse fibroblasts with a single factor, Oct4. Cell Rep.

[10] Lalit PA, Salick MR, Nelson DO (2016). Lineage reprogramming of fibroblasts into proliferative induced cardiac progenitor cells by defined factors. Cell Stem Cell.

[11] Zhang Y, Cao N, Huang Y (2016). Expandable cardiovascular progenitor cells reprogrammed from fibroblasts. Cell Stem Cell.

[12] Yamashita JK (2016). Expanding reprogramming to cardiovascular progenitors. Cell Stem Cell.

[13] Vierbuchen T, Ostermeier A, Pang ZP (2010). Direct conversion of fibroblasts to functional neurons by defined factors. Nature.

[14] Sekiya S, Suzuki A (2011). Direct conversion of mouse fibroblasts to hepatocyte-like cells by defined factors. Nature.

[15] Song K, Nam YJ, Luo X (2012). Heart repair by reprogramming non-myocytes with cardiac transcription factors. Nature.

[16] Chen JX, Krane M, Deutsch MA (2012). Inefficient reprogramming of fibroblasts into cardiomyocytes using Gata4, Mef2c, and Tbx5. Circ Res.

[17] Protze S, Khattak S, Poulet C (2012). A new approach to transcription factor screening for reprogramming of fibroblasts to cardiomyocyte-like cells. J Mol Cell Cardiol.

[18] Jayawardena TM, Egemnazarov B, Finch EA (2012). MicroRNA-mediated in vitro and in vivo direct reprogramming of cardiac fibroblasts to cardiomyocytes. Circ Res.

[19] Addis RC, Ifkovits JL, Pinto F (2013). Optimization of direct fibroblast reprogramming to cardiomyocytes using calcium activity as a functional measure of success. J Mol Cell Cardiol.

[20] Christoforou N, Chellappan M, Adler AF (2013). Transcription factors MYOCD, SRF, Mesp1 and SMARCD3 enhance the cardio-inducing effect of GATA4, TBX5, and MEF2C during direct cellular reprogramming. PLoS One.

[21] Muraoka N, Yamakawa H, Miyamoto K (2013). MiR-133 promotes cardiac reprogramming by directly repressing Snai1 and silencing fibroblast signatures. EMBO J.

[22] Hirai H, Katoku-Kikyo N, Keirstead SA (2013). Accelerated direct reprogramming of fibroblasts into cardiomyocyte-like cells with the MyoD transactivation domain. Cardiovasc Res.

[23] Wang L, Liu Z, Yin C (2015). Stoichiometry of Gata4, Mef2c, and Tbx5 influences the efficiency and quality of induced cardiac myocyte reprogramming. Circ Res.

[24] Ifkovits JL, Addis RC, Epstein JA (2014). Inhibition of TGFβ signaling increases direct conversion of fibroblasts to induced cardiomyocytes. PLoS ONE.

[25] Zhou H, Dickson ME, Kim MS (2015). Akt1/protein kinase B enhances transcriptional reprogramming of fibroblasts to functional cardiomyocytes. Natl Acad Sci USA.

[26] Fu Y, Huang C, Xu X (2015). Direct reprogramming of mouse fibroblasts into cardiomyocytes with chemical cocktails. Cell Res.

[27] Zhao Y, Londono P, Cao Y (2015). High-efficiency reprogramming of fibroblasts into cardiomyocytes requires suppression of pro-fibrotic signalling. Nat Commun.

[28] Yamakawa H, Muraoka N, Miyamoto K (2015). Fibroblast growth factors and vascular endothelial growth factor promote cardiac reprogramming under defined conditions. Stem Cell Rep.

[29] Nam YJ, Song K, Luo X (2013). Reprogramming of human fibroblasts toward a cardiac fate. Proc Natl Acad Sci U S A.

[30] Fu JD, Stone NR, Liu L (2013). Direct reprogramming of human fibroblasts toward a cardiomyocyte-like state. Stem Cell Rep.

[31] Wada R, Muraoka N, Inagawa K (2013). Induction of human cardiomyocyte-like cells from fibroblasts by defined factors. Proc Natl Acad Sci U S A.

[32] Islas JF, Liu Y, Weng KC (2012). Transcription factors ETS2 and MESP1 transdifferentiate human dermal fibroblasts into cardiac progenitors. Proc Natl Acad Sci U S A.

[33] Blum B, Benvenisty N (2008). The tumorigenicity of human embryonic stem cells. Adv Cancer Res.

[34] Tsuji O, Miura K, Okada Y (2010). Therapeutic potential of appropriately evaluated safe-induced pluripotent stem cells for spinal cord injury. Proc Natl Acad Sci U S A.

[35] Muraoka N, Ieda M (2014). Direct reprogramming of fibroblasts into myocytes to reverse fibrosis. Annu Rev Physiol.

[36] Qian L, Huang Y, Spencer CI (2012). In vivo reprogramming of murine cardiac fibroblasts into induced cardiomyocytes. Nature.

[37] Inagawa K, Miyamoto K, Yamakawa H (2012). Induction of cardiomyocyte-like cells in infarct hearts by gene transfer of Gata4, Mef2c, and Tbx5. Circ Res.

[38] Mathison M, Gersch RP, Nasser A (2012). In vivo cardiac cellular reprogramming efficacy is enhanced by angiogenic preconditioning of the infarcted myocardium with vascular endothelial growth factor. J Am Heart Assoc.

[39] Sadahiro T, Yamanaka S, Ieda M (2015). Direct cardiac reprogramming: progress and challenges in basic biology and clinical applications. Circ Res.

[40] Doppler SA, Deutsch MA, Lange R (2015). Direct reprogramming—the future of cardiac regeneration?. Int J Mol Sci.

[41] Yamakawa H, Ieda M (2015). Strategies for heart regeneration: approaches ranging from induced pluripotent stem cells to direct cardiac reprogramming. Int Heart J.

[42] Cao N, Huang Y, Zheng J (2016). Conversion of human fibroblasts into functional cardiomyocytes by small molecules. Science.

[43] Li XH, Li Q, Jiang L (2015). Generation of functional human cardiac progenitor cells by high-efficiency protein transduction. Stem Cells Transl Med.

